# Phosphate Burden and Organ Dysfunction

**DOI:** 10.3389/fragi.2022.890985

**Published:** 2022-07-14

**Authors:** Nikolay Mironov, Azeddine Atfi, Mohammed S. Razzaque

**Affiliations:** ^1^ Department of Pathology, Lake Erie College of Osteopathic Medicine, Erie, PA, United States; ^2^ Department of Pathology, Virginia Commonwealth University, Richmond, VA, United States

**Keywords:** phosphate, systemic effects, aging, calcification, kidney

## Introduction

Phosphorus is a ubiquitous mineral in nature and one of the most abundant minerals in the human body, representing about 1% of the total body weight (Calvo and Lamberg-Allardt, 2015). The body utilizes phosphorus in the form of phosphate (PO_4_). Phosphate maintains cellular membrane integrity, nucleic acid structure, generation of ATP, and key regulation of virtually every molecular pathway through phosphorylation or dephosphorylation of numerous enzymes and other proteins important for cell function and homeostasis. With so much utility, the body needs to maintain blood phosphate concentration at 2.5–4.5 mg/dl. The body maintains phosphate homeostasis via crosstalk among bone, kidney, and intestine. Phosphate enters the extracellular fluid pool and constantly moves in and out of bone to meet the body’s needs (Razzaque and Lanske, 2007; Razzaque, 2009a; Penido and Alon, 2012). Bones are a major phosphate reservoir, releasing it *via* the enzymatic activities of alkaline phosphatase. Alkaline phosphatase is found on the outer portion of the cell membrane and is responsible for catalyzing hydrolysis of organic phosphate esters present in extracellular space, allowing for intracellular movement of phosphate (Penido and Alon, 2012). The kidneys also regulate phosphate homeostasis, with most reabsorption occurring at the proximal tubule. The rate-limiting step of this reabsorption is mediated by two type II transporters: sodium-dependent phosphate cotransporter (NaPiIIa and NaPiIIc), located on apical membranes of proximal tubule cells where these cells reabsorb a total of 80% of filtered phosphate (Penido and Alon, 2012). NaPiIIa reabsorbs about 50% of filtered phosphate load, and its expression is partly regulated by parathyroid hormone (PTH), fibroblast growth factor 23 (FGF23), and dietary phosphate levels (Penido and Alon, 2012). NaPiIIc reabsorbs about 30% of proximal tubule phosphate and is regulated by FGF23, metabolic acidosis, dietary magnesium, and phosphate (Penido and Alon, 2012). Intestines absorb phosphate through various cellular and paracellular pathways, including passive diffusion, load-dependent processes, and active transport. Intestines regulate how much phosphate is absorbed into the bloodstream, and this process is controlled partly by vitamin D (Penido and Alon, 2012). Vitamin D-regulated expression of NaPiIIb brings phosphate into enterocytes via secondary active transport (Penido and Alon, 2012). PTH, by influencing the synthesis of vitamin D, indirectly regulates phosphate absorption in the duodenum and jejunum (Penido and Alon, 2012).

Numerous hormones are also involved in maintaining systemic phosphate homeostasis (Razzaque, 2022a). PTH acts on the kidneys to decrease phosphate reabsorption and increases the production of 1α-hydroxylase, which catalyzes the hydroxylation of calcifediol into calcitriol (the bioactive form of vitamin D). Increased production of 1,25(OH)_2_D_3_ (active vitamin D) enhances both calcium and phosphate absorption in the gut and also determines the extent of phosphate reabsorbed in the proximal tubule of the kidney via suppressing PTH activity (Penido and Alon, 2012). FGF23 increases renal excretion of phosphate and inhibits the synthesis of 1,25(OH)_2_D_3_ in attempts to lower serum phosphate concentrations (Prié and Friedlander, 2010; Agoro et al., 2020; Akimbekov et al., 2022; Nakatani et al., 2022). Renal excretion is accomplished by decreasing NaPiIIa and NaPiIIc protein expression levels (Prié and Friedlander, 2010). For FGF23 to be functional and lower serum phosphate levels, it needs Klotho, which increases FGF23’s affinity to its receptor at the target organs (Urakawa et al., 2006). Klotho is a single-pass transmembrane protein in the renal tubules, parathyroid gland, brain, and skeletal muscle (Prié and Friedlander, 2010). Klotho acts as an obligate cofactor for FGF23 binding and activation of cognate FGF receptors (Urakawa et al., 2006). The absence of FGF23 or Klotho leads to hyperphosphatemia and resulting premature aging features in mice (Razzaque et al., 2006; Nakatani et al., 2009a; Nakatani et al., 2009b). These features include but are not limited to loss of body weight, kyphosis, hypogonadism, infertility, generalized tissue atrophy, and reduced life span (Ohnishi and Razzaque, 2010), many of these alteration parallel potential aging sequelae.

### Phosphate Burden and Inflammation

Recent studies have found that phosphate burden can lead to the activation of inflammatory responses to propagate gingival inflammation and dental decay among children (Goodson et al., 2017; Goodson et al., 2019; Erem et al., 2022; Michigami et al., 2022). An increased salivary phosphate concentration has been associated with higher inflammatory markers and could predict childhood obesity (Hartman et al., 2013; Razzaque, 2022b). Hyperphosphatemia has associations with immune dysfunction. According to Plantinga et al., high phosphate levels early during dialysis were associated with an increased risk of infection when adjusting for secondary hyperparathyroidism, uremia, or poor dialysis (Plantinga et al., 2008). Patients with end-stage renal disease (ESRD) poorly respond to immunizations against pathogens, have impaired cell-mediated immunity, and reduced CD4^+^/CD8^+^ T lymphocyte ratio (Plantinga et al., 2008). Decreases in T lymphocyte numbers are likely due to increased oxidative stress and accumulation of uremic toxicity, both are features of ESRD (Plantinga et al., 2008). Investigators also discovered a negative correlation between hyperphosphatemia severity and a number of naive subsets of T lymphocytes, raising the possibility that hyperphosphatemia plays a role in reduced numbers of T cells seen in ESRD (Plantinga et al., 2008).

One of the many parameters categorizing aging is the functional decline of the healthy immune system, leaving the older population more susceptible to pathogens causing bacterial pneumonia and influenza (Sosa et al., 2020). Sosa et al. found pro-inflammatory cytokine expression higher in aged mice, with 40% serum phosphate levels beyond those of their counterparts. These cytokine values were decreased when they were fed a low phosphate diet (Sosa et al., 2020). They also found a positive correlation between Interleukin-1β (IL-1β) expression and serum phosphate levels, effectively showing hyperphosphatemia increases inflammation *in vivo* (Sosa et al., 2020). IL-1β is an essential factor for acute host responses and resistance to pathogens, while exacerbating damage during chronic disease and acute injury (Lopez-Castejon and Brough, 2011). Dietary phosphate loading promotes systemic inflammation and oxidative stress measured by serum Tumor necrosis factor alpha (TNF-α) and urinary 8-hydroxy-2′-deoxyguanine to creatinine (8-OHdG/Cr) levels; 8-OHdG is a metabolite of DNA repair and is measurable in the urine (Yamada et al., 2014). TNF-α is released by macrophages or monocytes and has many functions including necrosis or inflammation (Besse et al., 2022). Dietary phosphate loading increases TNF-α in a dose-dependent fashion; serum TNF-α levels were significantly correlated with urinary 8-OHdG/Cr levels (a measure of oxidative stress) (Yamada et al., 2014). The investigators also observed a decrease in TNF-α and OHdG/Cr when lowering dietary phosphate intake or reducing serum phosphate levels (Yamada et al., 2014). This may also be one way to explain the chronic low grade of inflammation found in the elderly with altered phosphate balance. Although intermittent inflammation is needed for survival during infections and physical injury, chronic systemic inflammation is detrimental to human health (Pinti et al., 2014). Chronic inflammation increases the incidence of many diseases in the elderly, such as cardiovascular disease (CVD), cancer, diabetes mellitus, chronic kidney disease (CKD), autoimmune, and neurodegenerative disorders (Furman et al., 2019). High phosphate burden also increases inflammatory responses in experimental studies, as demonstrated by Yamada et al., who found that increasing phosphate loads in the body led to increased mRNA levels of TNF-α in the aorta, heart, and kidney in rats (Yamada et al., 2014). Cancer patients are also known to experience increased phosphate burden compared to non-cancer patients, most likely due to increased metabolic activity of cancer cells. Elevated phosphate burdens have been shown to positively correlate with the risk of lung, pancreas, thyroid, and bone cancers in men, and cancers of the esophagus, lung, and nonmelanoma skin cancer in women (Brown and Razzaque, 2018). Studies have shown that elevated phosphate levels can induce epithelial to mesenchymal transition (EMT), a major cellular event related to tumor invasion and metastasis (He et al., 2021; Alexander et al., 2022; Lewis et al., 2022).

### Phosphate Burden and Aging

Aging is a complex biological process where progressive accumulation of age-associated changes with time are associated with or directly responsible for the increased susceptibility to disease and death, which accompanies advancing age (Boss and Seegmiller, 1981; Ohnishi and Razzaque, 2010). It decreases cardiac output by 1% a year after 30, mostly due to reduced response of catecholamines and cardiac glycosides by cardiac muscle cells (Boss and Seegmiller, 1981). Blood pressure increases as there is progressive stiffening of arteries with age, particularly in the aorta, increasing afterload or the load against which the heart has to contract to eject blood (Boss and Seegmiller, 1981). Natural lipid deposits in vessels increase the risk for arteriosclerosis and coronary artery disease. Decreases in lung volume and elastic recoil leads to an increase in residual volume, which is the volume of air that cannot be exhaled from the lungs (Boss and Seegmiller, 1981). Decreasing elastic recoil causes a greater tendency for airways to collapse, resulting in ventilation-perfusion mismatches (Boss and Seegmiller, 1981). Kidney size and glomeruli number decrease by about 30% by age 65 (Boss and Seegmiller, 1981). Many of these age-associated changes are not pathologic but stack the odds towards pathology.

Hyperphosphatemia is most often caused by renal failure, as the kidneys excrete up to 90% of daily phosphate, leaving the other 10% to the gut (Goyal and Jialal, 2022). High phosphate levels are caused by its high intake, vitamin D intoxication, and several genetic diseases. Potential symptoms associated with hyperphosphatemia are hypocalcemia due to calcium-phosphate precipitation in the skin and soft tissues, vascular calcifications, and arteriosclerosis. High phosphate levels manifest with central nervous system disturbances such as coma, seizures, delirium, neuromuscular excitability, muscle cramping, tetany, and eventual cognitive decline (Acquaviva et al., 2022; Goyal and Jialal, 2022). It leads to cataracts and conjunctivitis in the eye from induction of symptomatic hypocalcemia due to calcium-phosphate precipitation (Goyal and Jialal, 2022). Renal failure results in reduced synthesis of calcitriol and secondary hyperparathyroidism, causing increased osteoclastic bone reabsorption and release of calcium and phosphate into the circulation and this lengthened bone demineralization leads to increased occurrences of fractures (Goyal and Jialal, 2022). Hyperphosphatemia induces changes in endothelial cells, such as declines in nitric oxide (NO) production due to oxidative stress, thereby leading to reduced cell viability and increased apoptosis (Peng et al., 2011). High phosphate levels lead to endothelial cell senescence via cell cycle arrest, thereby leading to senescence rather than death via apoptosis (Terzi et al., 2016; Olmos et al., 2017; Maique et al., 2020; Hu and Moe, 2022).

Aging is a process that is characterized by increased susceptibility of individuals, as they age, to factors that eventually lead to their morbidity and mortality (Weinert and Timiras, 2003; Jayanthi et al., 2010). As individuals age, they have progressive loss of tissue and organ functions, leading to the development of the oxidative stress theory of aging (OSTA) hypothesis. OSTA suggests the aging rate is directly related to the accumulation of oxidative damage (Salmon et al., 2010). It is based on structural damage resulting from the accumulation of oxidative damage to macromolecules (DNA, protein) via reactive oxygen (ROS) and nitrogen (RONS) species. High ROS levels over a long period activate signaling pathways, which accelerate proteolysis and eventual cell death (Barreiro, 2016). In a study performed by Nagai et al. using Klotho deficient mice, the investigators demonstrated that hyperphosphatemia resulted in cognition impairment due to increased oxidative damage and apoptosis in hippocampus neurons, which could be rescued by administering an antioxidant (Nagai et al., 2003).

Hyperphosphatemia leads to extensive oxidative stress in the mitochondria, although it is unclear how phosphate increases ROS generation and mitochondrial permeability transition (MPT). The most conceivable hypothesis is that phosphate catalyzes reactions that favor ROS formation (Kowaltowski et al., 2001). MPT is one of the ways mitochondria release apoptotic signal molecules into the cytosol. MPT causes non-selective increased permeability of the inner mitochondrial membrane resulting in loss of matrix components, swelling, and eventual rupture and cytochrome C release (Zoratti and Szabò, 1995). Zhao et al. have found that hyperphosphatemia induced calcification with oxidative stress of mitochondria (Zhao et al., 2011). A decline in mitochondrial function has long-held associations with an increase in features of aging (McGuire, 2019). Such changes lead to programmed cell death or apoptosis. Long-term exposure to high phosphate levels potentiates aging and age-related disorders (Ohnishi and Razzaque, 2010; Jacob et al., 2013; He et al., 2021; Hetz et al., 2021).

### Phosphate Burden and Cardiovascular Pathology

Hyperphosphatemia impairs endothelial cell function through endothelin-1 and NO imbalances leading to dysfunction of the endothelium, an important step in the pathogenesis of atherosclerosis which can impair functionality of all the organs, including renal and cardiac functions (Olmos et al., 2017). High phosphate levels caused a decline in NO production *via* bradykinin and increased ROS, thereby leading to endothelial dysfunction (Peng et al., 2011). Hyperphosphatemia also reduced intracellular calcium levels, increased protein kinase C-B_2_, increased apoptosis, and reduced cell viability (Peng et al., 2011). Hyperphosphatemia accelerated vascular aging by collagenization of the tunica media in the walls of arteries, with phosphate and calcium crystals accumulating in the elastic fibers of the vessel (Boss and Seegmiller, 1981).

**FIGURE 1:**
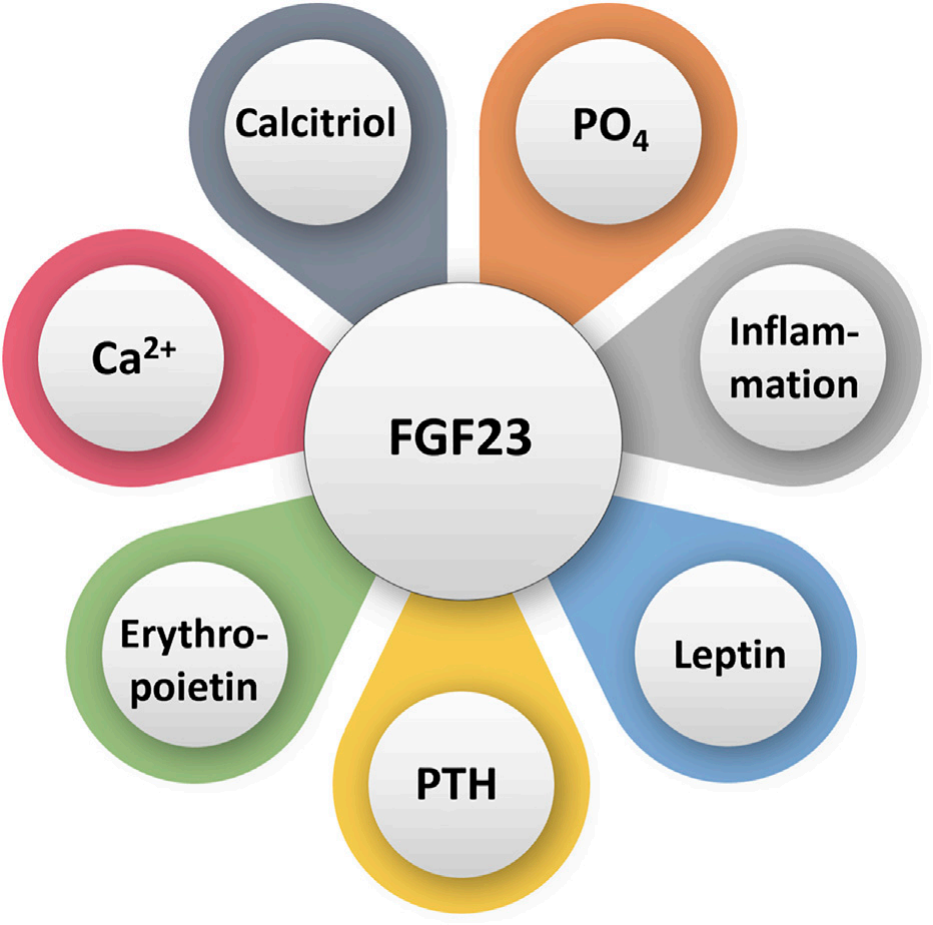
Simplified diagram illustrating various factors that can directly or indirectly influence FGF23 activities. PO_4_: phosphate; Ca^2+^: calcium.

FGF23 is a hormone that lowers blood phosphate levels (Razzaque and Lanske, 2007; Razzaque, 2009a; Razzaque, 2009b). When phosphate levels are high, bone secretes FGF23, which acts on the kidney to increase the excretion of phosphate (Pinti et al., 2014) (Figure 1). FGF23 also suppresses vitamin D synthesis by inhibiting cytochrome P27B1 and stimulating cytochrome P24 to reduce the levels of 1,25(OH)_2_D_3_ (Pinti et al., 2014). Vitamin D increases the absorption of calcium and phosphate in the intestines and mobilizes bone tissue via enhancing osteoclastic activities to increase blood levels of phosphate and calcium (Furman et al., 2019). Severe hyperphosphatemia is induced in human diseases where FGF23 is mutated (Benet-Pagès et al., 2005; Onishi et al., 2008; Chakhtoura et al., 2018). FGF23 gene deletion from mice resulted in hyperphosphatemia (Sitara et al., 2004), thus solidifying the role of FGF23 in reducing serum phosphate levels. The mouse with nonfunctioning FGF23 had lower lifespan than the wild-type counterparts. This decrease in lifespan was partially due to generalized tissue and organ atrophy and vascular calcifications. Hyperphosphatemic mice also had lower adipose and skeletal muscle mass than mice with normal phosphate levels, further attesting to the accelerated aging in these mice (Sitara et al., 2004). Finally, FGF23 deficient mice were also infertile, hypoglycemic, and had increased total serum cholesterol (Shimada et al., 2004; Sitara et al., 2004).

Another gene that regulates phosphate homeostasis and is critical for FGF23 function is Klotho. Both Klotho and FGF23 deficient mice consistently display signs of premature aging and CKD-associated with mineral and bone disorders. Klotho deficient mice are completely resistant to FGF23 and thus develop hyperphosphatemia (Nakatani et al., 2009a). Some of the symptoms seen in these mice have shorter lifespan, infertility, arteriosclerosis, skin atrophy, and emphysema (Nakatani et al., 2009b; Ohnishi and Razzaque, 2010). It is important to note that the Klotho deficient mice could be rescued from all symptoms by reducing phosphate levels towards the normal ranges (Ohnishi and Razzaque, 2010). CKD and its complications, such as vascular calcification, CKD-mineral and bone disease (MBD), all result from a Klotho deficiency, which manifests as accelerated aging due to phosphate burden.

Hyperphosphatemia leads to vascular dysfunction through endothelin 1 and NO imbalances. A study by Foley et al. have found evidence for hyperphosphatemia increasing incidence of cardiac calcification, left ventricular hypertrophy, and cardiovascular events, including deaths, were all accelerated with hyperphosphatemia (Foley, 2009; Foley et al., 2009). Although the reason is not entirely mapped out yet, one thought could be via a mechanism other than vascular calcification. It could be Klotho or FGF23 dysfunctions, which, as previously discussed, have shown to lead to CVD in mice. Hyperphosphatemia changes the amount of Klotho, FGF23, PTH, and calcitriol in the body, therefore increasing CVD incidence (Foley, 2009). Hyperphosphatemia increases CVD risk in individuals who do not have CKD and CVD (Dhingra et al., 2007). Phosphate burden (higher than 3.5 mg/dl) was associated with 55% increased CVD risk. This could be because high phosphate levels inhibit vitamin D synthesis; low levels of vitamin D have been hypothesized to decrease cardiac contractility and vascular dysfunction (Dhingra et al., 2007). High phosphate levels have also been found to induce endothelial cell dysfunction via lowering NO levels and intracellular calcium levels, and attendant apoptosis and reduced cell viability (Olmos et al., 2017). It is important to note that sevelamer carbonate, a phosphate scavenger, improved endothelial function and reduced mortality in patients with type 2 diabetes mellitus and inflammation in patients on peritoneal dialysis for kidney failure (Chennasamudram et al., 2013). Sevelamer, which binds phosphate in gut and prevents absorption, improved endothelial function and decreased plasminogen activator inhibitor 1, C-reactive protein, and IL-6 (Chennasamudram et al., 2013). Dysfunction of endothelial cells has been associated with the development of cardiovascular and renal damage in diabetes, hypertension, or atherosclerosis (Peng et al., 2011; Chennasamudram et al., 2013). A high phosphate burden increases oxidative stress in endothelial cells leading to cellular dysfunction. When dysfunctional, endothelial cells are unable to synthesize nitric oxide, aggravating atherosclerotic plaque formation occurs in Apo-E deficient mice (Shiota et al., 2011). A high-phosphate diet accelerated atherogenesis in Apo-E deficient mice (Ellam et al., 2011).

### Phosphate Burden and Muscular Pathology

A high phosphate burden accelerates skeletal muscle atrophy through mechanisms not fully understood yet. Chung et al. demonstrated increased phosphate levels leading to increased muscle wasting owing to reduced myotubule size, increased ROS generation, decreased protein synthesis, and accelerated protein degradation (Chung et al., 2020). This is especially important with aged individuals, as their musculoskeletal system tends to breakdown with increase in age. Aging is associated with progressive and involuntary loss of muscle mass and strength, a condition known as sarcopenia. Sosa et al. have found hyperphosphatemia induces cellular senescence in murine myoblasts, leading to sarcopenia as one potential consequence (Sosa et al., 2018; Sosa et al., 2021). Of relevance, cellular senescence is the inability to progress through the cell cycle. This occurred to myoblasts due to increased mTOR activation and reduced autophagy under hyperphosphatemia conditions via Integrin-linked kinase (ILK) activation (Sosa et al., 2018), which is essential to myoblast senescence; suppressing ILK expression resulted in increased autophagy and protected myoblasts from senescence triggered by hyperphosphatemia (Sosa et al., 2018). With myoblast losing their proliferative abilities, sarcopenia may develop (Sosa et al., 2018). This was identified through hyperphosphatemia inducing senescence in cultured myoblasts through ILK overexpression via gene transfer using adenoviral expression vectors encoding ILK gene, lowers cell replication capacity since older mice have a considerable loss of muscle strength, which correlates with hyperphosphatemia and increased ILK and p53 (Sosa et al., 2018). Overexpression of ILK upregulates p53, which is a cell cycle inhibitor (Sosa et al., 2018). It is also important to discuss CKD and its role in accelerating muscle loss. Muscle atrophy is a major clinical issue in CKD patients, and muscle preservation has an integral part in the patient treatment and outcomes (Chung et al., 2020). A high phosphate burden has been suggested to suppress myogenic differentiation *in vitro* and promote skeletal muscle atrophy *in vivo* in diseases such as CKD. This is mainly accomplished through enhanced nuclear factor erythroid 2-related factor 2 (Nrf2) transcriptional activity via increased ROS generation and p62 expression (Chung et al., 2020). Nrf2 is a sensor of oxidative stress and is prevented from binding to DNA by Kelch-like ECH-associated protein 1 (Keap1). Keap1 is inactivated during oxidative stress which allows Nrf2 to influence multiple mechanisms including drug metabolism, oxidant signaling, and antioxidant defense (Chung et al., 2020). P62 is a stress induced protein which leads to inclusion body formations and can also target ubiquitinated proteins for digestion (Chung et al., 2020). Experimental animal studies have shown that hyperphosphatemia increases inflammation to intensify anemia and skeletal muscle wasting (Czaya et al., 2022); phosphate burden induces hepatic levels of IL-6 and IL-1β to enhance the expression of hepcidin, a potential causative link between hyperphosphatemia, anemia, and skeletal muscle dysfunction (Czaya et al., 2022). Hepcidin regulates systemic iron homeostasis by blocking intestinal iron absorption and macrophage iron recycling at high levels (Czaya et al., 2022).

### Phosphate Burden and Renal Pathology

CKD is associated with hyperphosphatemia, which increases the odds of developing various diseases, such as coronary artery disease (John et al., 2011). As mentioned above, kidneys are responsible for phosphate excretion to keep levels in an optimal range. CKD leads to increased numbers of nonfunctioning nephrons as well as increased amounts of phosphate in the body (Foley, 2009). CKD does not allow for successful aging, which is desirable by most. Successful aging is defined as aging while remaining free of CVD, cancer, chronic obstructive pulmonary disease (COPD), and personal/cognitive disability or impairment (Sarnak et al., 2008). Sarnak et al. have found that impaired renal function, such as CKD, promotes unsuccessful aging (Sarnak et al., 2008). Although not completely understood why CKD promotes unsuccessful aging, Sarnak et al. proposed three potential mechanisms. First, kidney dysfunction may be the secondary symptom due to vascular disease or hypertension. Second, kidney function may mediate an increase in several other risk factors for aging, like anemia, insulin resistance, and inflammation. Third, kidney dysfunction may be linked to unsuccessful aging related to insufficient glomerular filtration rate (GFR) (Sarnak et al., 2008). Even early stages of CKD can drop a minimum of 5 years to the normal life span (Sarnak et al., 2008). CKD culminates in systemic mineral metabolism and bone composition along with a decrease in GFR (Hou et al., 2018). This creates a scenario known as CKD-MBD. With falling GFR levels, serum calcium and phosphate levels rise (Hou et al., 2018). Disruption in mineral homeostasis increases secretion of PTH, FGF23, and decreases calcitriol. These effects combined lead to increased bone turnover and extra-skeletal calcifications (Sprague et al., 2021). Hyperphosphatemia, vascular calcification, and elevated FGF23 concentrations are the components of CKD-MBD, which exacerbate cardiovascular disease, accounting for around 60% of deaths among patients with CKD on dialysis (Sprague et al., 2021).

## Conclusion

**FIGURE 2:**
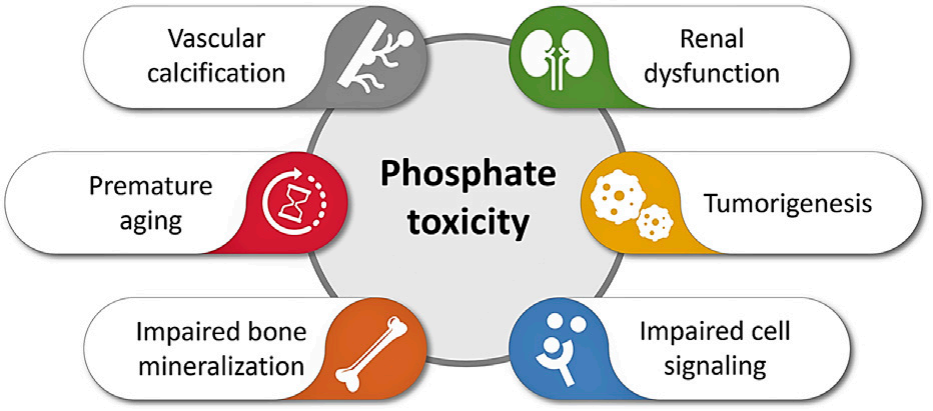
Simplified diagram illustrating various adverse events of phosphate toxicity, ranging from impaired bone mineralization to vascular calcification to accelerated aging (inspired based on earlier publications) (Akimbekov et al., 2022; Razzaque, 2022a; Erem et al., 2022; Michigami et al., 2022; Nakatani et al., 2022).

Phosphate is an important nutrient that has various roles in the human body. It is imperative to keep its concentration in normal homeostatic ranges to avoid increasing chances of developing numerous systemic pathologies, as discussed earlier. Hyperphosphatemia has a role in many aspects of accelerated aging, prominent among them sarcopenia, decreased immune function, skin atrophy, development of arteriosclerosis, tumorigenesis, or the progression of various neurodegenerative disorders (Figure 2). Potential interventions to delay phosphate-associated aging-like features could be through decreasing phosphate burden with phosphate scavengers. Reducing dietary phosphate intake is another intervention, which could be achieved through avoiding artificially added phosphate-rich processed foods (Miyamoto et al., 2022). As phosphate is commonly found in additives and preservatives, the FDA does not require food industries to list amounts of phosphate on labels, thus making the task of controlling the amount of consumed much more challenging. In closing, it is becoming increasingly clear that hyperphosphatemia represents a major driver of accelerated aging, emphasizing the unmet needs for further interventional studies with the potential to yield therapeutic breakthroughs.
